# Antimycobacterial and cytotoxic activity of selected medicinal plant extracts

**DOI:** 10.1016/j.jep.2016.02.010

**Published:** 2016-04-22

**Authors:** Joseph M. Nguta, Regina Appiah-Opong, Alexander K. Nyarko, Dorothy Yeboah-Manu, Phyllis G.A. Addo, Isaac Otchere, Abena Kissi-Twum

**Affiliations:** a Department of Clinical Pathology, Noguchi Memorial Institute for Medical Research, University of Ghana, Ghana; b Department of Public Health, Pharmacology and Toxicology, Faculty of Veterinary Medicine, University of Nairobi, Kenya; c Department of Bacteriology, Noguchi Memorial Institute for Medical Research, University of Ghana, Ghana; d Department of Animal Experimentation, Noguchi Memorial Institute for Medical Research, University of Ghana, Ghana

**Keywords:** Tuberculosis, Antimycobacterial, Cytotoxicity, Selectivity, Medicinal plants, Crude extracts

## Abstract

**Ethnopharmacological relevance:**

Tuberculosis (TB) caused by *Mycobacterium tuberculosis* remains an ongoing threat to human health. Several medicinal plants are used traditionally to treat tuberculosis in Ghana. The current study was designed to investigate the antimycobacterial activity and cytotoxicity of crude extracts from five selected medicinal plants.

**Material and methods:**

The microplate alamar blue assay (MABA) was used for antimycobacterial studies while the CellTiter 96® AQ_ueous_ Assay, which is composed of solutions of a novel tetrazolium compound [3-(4,5-dimethylthiazol-2-yl)-5-(3-carboxymethoxyphenyl)-2-(4-sulfophenyl)-2H-tetrazolium, inner salt; MTS] and an electron coupling reagent (phenazine methosulfate) PMS, was used for cytotoxic studies. Correlation coefficients were used to compare the activity of crude extracts against nonpathogenic strains and the pathogenic *Mycobacterium tuberculosis subsp*.*tuberculosis.*

**Results:**

Results of the MIC determinations indicated that all the crude extracts were active on all the three tested mycobacterial strains. Minimum inhibitory concentration values as low as 156.3 µg/mL against *M. tuberculosis*; Strain H37Ra (ATCC® 25,177™) were recorded from the leaves of *Solanum torvum* Sw. (Solanaceae). Cytotoxicity of the extracts varied, and the leaves from *S. torvum* had the most promising selectivity index. Activity against *M. tuberculosis*; Strain H37Ra was the best predictor of activity against pathogenic *Mycobacterium tuberculosis subsp*.*tuberculosis* (correlation coefficient=0.8).

**Conclusion:**

The overall results of the present study provide supportive data on the use of some medicinal plants for tuberculosis treatment. The leaves of *Solanum torvum* are a potential source of anti-TB natural products and deserve further investigations to develop novel anti-TB agents against sensitive and drug resistant strains of *M. tuberculosis*.

## Graphical abstract

**Figure:**
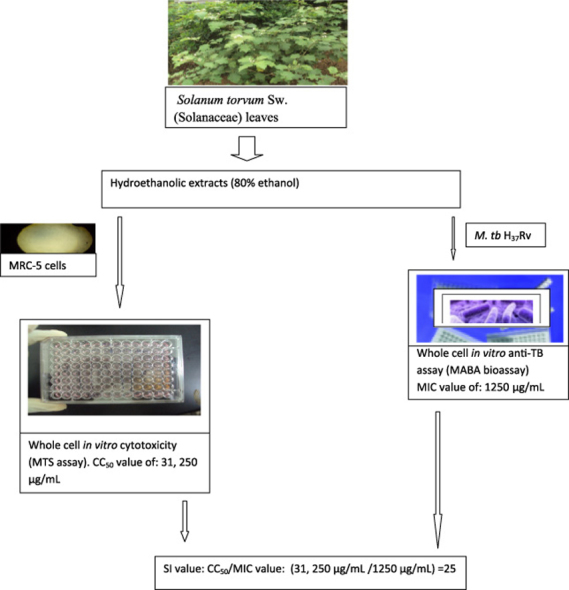

## 1 Introduction

*Mycobacterium tuberculosis*, a facultative intracellular microbe belonging to the *M. tuberculosis* complex, is the most important cause of tuberculosis (TB) in humans. In addition to *M. tuberculosis*, other members of the *M. tuberculosis* complex that are able to cause tuberculosis in humans include *Mycobacterium bovis*, *Mycobacterium africanum*, *Mycobacterium microti* and *Mycobacterium canetti* (Aro et al., 2015). Tuberculosis, an old yet emerging infectious disease, is one of the leading causes of human morbidity and mortality (Nguta et al., 2015b). In the year 2013, the World Health Organization (WHO) reported one and half million deaths and nine million new cases of active tuberculosis globally caused by TB (WHO, 2014). The alarming rise of multi-drug-resistant (MDR), extensively drug-resistant (XDR) and currently, totally drug resistant (TDR) *M. tuberculosis* strains, which are difficult to control with the currently available essential antitubercular drugs on the market, and the increased incidence of TB associated with viral infections such as HIV, have recently complicated the chemotherapeutics of tuberculosis (Proksch et al., 2015). Thus, the search for new drugs with novel mechanisms of action against *M. tuberculosis* is urgently needed.

Historically, natural products have proved to be the most prolific and diverse source of antibiotics including some of those used for the treatment of TB. Current studies have indicated the urgent need for the development of new, safe and efficacious drugs to help reduce the global burden of tuberculosis. Novel antimycobacterial scaffolds from natural products have recently been reported. Natural products of plant biodiversity have received considerable attention as potential anti-TB agents since they are a proven template for the development of new molecules against tuberculosis. Many antitubercular compounds that may prove to be useful leads for TB drug discovery have been derived from medicinal plants (Nguta et al., 2015b).

Natural products, especially those from the plant biodiversity have been less intensively investigated in the past even though they are known to contain structurally diverse molecules, many of which are unknown. This has prompted us to investigate Ghanaian medicinal plants for their anti-TB activity. In this study, we examined the leaves of *Solanum torvum* Sw. (Solanaceae), *Aloe vera var*. *barbadensis (*Xanthorrhoeaceae), *Dissotis rotundifolia* (Sm.) Triana (Melastomataceae), *Chenopodium ambrosioides* L. (Amaranthaceae) and the rhizomes of *Zingiber officinale* Roscoe *(Zingiberaceae),* all obtained from the eastern region of Ghana. These medicinal plants have been traditionally used by Ghanaian communities to treat coughs and other disease conditions with symptoms of tuberculosis (Nguta et al., 2015a). *S. torvum* is a shrub widely distributed in South India, Malaysia, China, Philippines, Thailand, West Indies and Tropical Africa. Antimicrobial activities of the leaf and fruit of this plant have been reported. The fruits of *S. torvum* are edible and traditionally used for the treatment of abscesses, jigger wounds, skin infections and athlete’s foot (Chandrasekhar et al., 2012). Ginger (*Z. officinale)* is a medicinal plant that has been widely used in Chinese, Ayurvedic and Tibb-Unani herbal medicines all over the world, since antiquity, for a wide array of unrelated ailments that include arthritis, rheumatism, sprains, muscular aches, pains, sore throats, cramps, constipation, indigestion, vomiting, hypertension, dementia, fever, infectious diseases and helminthiasis (Ali et al., 2008). *Aloe vera* is as old as civilization and throughout history it has been used as a popular folk medicine. It is present in the arid regions of India and is believed to be effective in treating stomach ailments, various skin conditions such as cuts, burns and eczema, gastrointestinal problems, skin diseases, constipation, anti-inflammatory effects, wound healing, as an anti-ulcer and anti-diabetic agent. Currently the plant is widely used in skin care, cosmetics and as a neutraceutical (Klein and Penneys, 1988). *D. rotundifolia* and *C. ambrosioides* are traditionally used in Ghanaian ethnomedicine against upper and lower respiratory tract conditions (Nguta et al., 2015a).

All the plant species were evaluated *in vitro* against *M.tuberculosis subsp*.*tuberculosis* (ATCC® 27,294™), *M. tuberculosis*; Strain H37Ra (ATCC® 25,177™) and *Mycobacterium smegmatis* (ATCC®19420™) for anti-TB activity as well as against MRC-5, human fetal lung fibroblast cell lines (ATCC® CCL-171™) in order to assess their general cytotoxicity. The study was extended to further investigate the best predictor of activity against pathogenic *M. tuberculosis subsp*.*tuberculosis* from the non-pathogenic strains tested.

## 2 Materials and methods

### 2.1 Antibiotics and chemicals

Isoniazid>99%, (INH), rifampicin 95% (RIF) and ethambutol hydrochloride, European pharmacopoeia (EP) reference standard (EMB) were obtained from Sigma-Aldrich (St. Louis, Mo.). Stock solutions at 1000 μg/mL were filter sterilized and stored at −20 °C. Working solutions were prepared at four times the final higher concentration in 7H9GC-Tween (Middlebrook 7H9 supplemented with 0.1% casitone, 0.5% glycerol, 10% OADC (oleic acid, albumin, dextrose, and catalase); Becton-Dickinson and 0.05% (vol/vol) Tween 80 (Sigma). The final drug concentrations tested were as follows: for crude extracts, 10,000 μg/mL; INH, 1.6 μg/mL; and for RIF and EMB, 16 μg/mL. A mixture of 10X Alamar blue dye (Alamar Biosciences/Accumed, Westlake, Ohio) and 10% Tween 80 (1:1) was sterilized by filtration, and stored at 4 °C for up to 1 week.

### 2.2 Collection and extraction of plant Materials

The leaves of *Solanum torvum, Aloe vera var*. *barbadensis, D. rotundifolia*, *C. ambrosioides* and the rhizomes of *Z. officinale* were all collected from the eastern region of Ghana during July-August in 2014 as earlier described (Nguta et al., 2015a). The plant materials were identified at the Centre for Scientific Research into Plant Medicine (CSRPM), Mampong-Akuapem, where voucher specimens are deposited. The plant materials were air dried at room temperature, ground and stored at 4 °C until use. The air dried and powdered plant parts each weighing 100 g were extracted with 500 mls of 80% ethanol (Sigma-Aldrich, Munich, Germany) by cold maceration at room temperature for 72 h to yield a powder after filtration and evaporation under vacuum. The dry powder was made up to a concentration of 10,000 μg/mL in distilled water, filter sterilized and stored at 20 °C until use.

### 2.3 *In vitro* antimycobacterial assay

#### 2.3.1 Bacterial strains and growth conditions

*Mycobacterium tuberculosis subsp*.*tuberculosis Mycobacterium tuberculosis*; Strain H37Ra and *Mycobacterium smegmatis* were obtained from the American Type Culture Collection (Manassas, VA 20,108, USA). They were maintained on Lowenstein-Jensen (LJ) slopes and cultured on Middlebrook 7H9 broth (Difco, Detroit, Mich.) supplemented with 0.2% (vol/vol) glycerol (Sigma Chemical Co., Saint Louis, Mo.), 1.0 g of casitone (Difco) per liter, 10% (vol/vol) OADC (oleic acid, albumin, dextrose, catalase; Difco) and 0.05% (vol/vol) Tween 80 (Sigma). The complete medium was referred to as 7H9GC-Tween. Cultures were grown aerobically on 7H9GC-Tween at 37 °C.

#### 2.3.2 Microplate alamar blue assay (MABA)

Mycobacterial strains were freshly sub cultured on LJ medium. The inoculum was prepared in 7H9GC-Tween broth, adjusted spectrophotometrically to a no. 1 McFarland tube standard, and further diluted 1:10 in 7H9GC-Tween broth for the test (Palomino et al., 2002).

The microplate alamar blue assay was carried out as described by Palomino et al. (2002). Briefly, 100 μL of 7H9GC-Tween broth was dispensed in each well of a sterile flat-bottom 96-well plate, and serial twofold dilutions of the crude extracts and each positive control drug were prepared directly in the plate. One hundred microliters of inoculum was added to each well. A growth control and a sterile control were also included for each mycobacterial strain. Sterile water was added to all perimeter wells to avoid evaporation during the incubation. The plates were covered, sealed in plastic bags, and incubated at 37 °C under a normal atmosphere. After 48 h of incubation for *M. smegmatis* and 7 days of incubation for *M. tuberculosis* H37Ra and *M. tuberculosis subsp*. *tuberculosis*, 30 μL of a mixture of alamar blue solution and 10% tween 80 (1:1) was added to each well, and the plate was reincubated overnight. A change in color from blue to pink indicated the growth of bacteria, and the minimum inhibitory concentration (MIC) was defined as the lowest concentration of the crude extract or positive control drug that prevented this change in color. The crude extract and drug concentration ranges used were as follows: for crude extracts, 10,000 to 19.5 μg/mL, INH, 0.025 to 1.6 μg/mL and EMB and RIF, 0.25 to 16 μg/mL. All the experiments were carried out in triplicate and were conducted in a Biosafety Level Three (BSL-3) laboratory.

### 2.4 Cytotoxicity assay

#### 2.4.1 Chemicals

*In vitro* assay reagents were purchased from Promega (USA), Sigma (USA) and Gibco (USA).

#### 2.4.2 Human fetal lung fibroblasts and culture conditions

MRC-5, human fetal lung fibroblast cell line was obtained from the American Type Culture Collection (Manassas, VA 20108, USA). All cultures were maintained in a phenol red free culture medium DMEM/F12 (Dulbecco’s modified essential medium/Ham’s 12 nutrient mixture, Gibco), supplemented with 5% (vol/vol) fetal calf serum (JS Bioscience, Australia), and 1% (vol/vol) antibiotic (2 mM l-glutamine, 100 U/mL Penicillin and 100 μg/mL Streptomycin; Gibco). Cultured cells were kept at 37 °C in a humidified 5% carbon dioxide (CO_2_) incubator. Once the cells reached confluence, the culture medium was removed from the flask and the cells were rinsed three times with sterile HBSS (Hank’s Balanced Salt Solution, Gibco). The confluent cell layers were enzymatically removed, using Trypsin/EDTA (Gibco, USA), and resuspended in culture medium. Cell viability was assessed by vital staining with trypan blue (0.4% (wt/vol); Sigma, USA), and cell numbers were determined using a light microscope (Leitz Wetzlar, Germany).

#### 2.4.3 Preparation of crude extracts

Crude extracts were individually suspended in the culture medium at the concentration of 10 000 μg/mL and dispersed by ultrasonic vibration for 15 min. In order to ensure uniform suspension, they were stirred on vortex agitation (1 min) before every use.

#### 2.4.4 Colorimetric MTS (3-(4, 5-dimethylthiazol−2-yl)-5-(3-carboxymethoxyphenyl)-2-(4-sulfophenyl)-2H tetrazolium) *in vitro* assay

Cytotoxicity testing was performed using the Promega CellTiter 96 AQueous Non-Radioactive Cell Proliferation (MTS) assay to determine the number of viable cells in culture (Promega, 2005). The protocol for evaluation of cytotoxicity was adopted from previously published papers and manufacturer’s instructions (Bakand et al., 2005a, Bakand et al., 2005b, Hayes et al., 2007). Crude extracts were suspended in culture media, serially diluted across 96-well microtiter plates (100 μL), and incubated at 37 °C with 5% CO_2_ for 24 h in a humidified carbon dioxide incubator. Four hours prior to the end of each exposure period, an MTS mixture (20 μL/well) was added. After the completion of exposure period, the plates were then placed on an Infinite M200 Pro™ plate reader (Tecan, Austria, GmbH), shaken for ten seconds and the absorbance of the formazan product was read at 490 nm. Each experiment was repeated on three separate occasions. Two internal controls were set up for each experiment: (1) an IC_0_ consisting of cells only and (2) IC_100_ consisting of medium only. Background absorbance due to the non-specific reaction between crude extracts and the MTS reagent was deducted from exposed cell values (Hayes and Markovic, 2002).

#### 2.4.5 Dose response curves of *in vitro* cytotoxicity data

Dose response curves were plotted for the crude extracts after correction by subtracting the background absorbance from the controls. The percentage inhibition (%I) was determined using the formula:

%I=[100×(1-At/Ac)

where (At) is the absorbance of treated well and (Ac) is the absorbance of control well. CC_50_ value is the concentration of sample required to inhibit 50% of the cell proliferation and was calculated from a calibration curve by a linear regression (Joshi et al., 2010) using Microsoft Excel. The selective index was determined as the ratio CC_50_/MIC value.

## 3 Results and discussion

In the present study, we examined the *in vitro* antimycobacterial activity of the leaves of *Solanum torvum Aloe vera var*. *barbadensis*, *Dissotis rotundifolia, Chenopodium ambrosioides* and the rhizomes of *Zingiber officinale* Roscoe against slow growing pathogenic *Mycobacterium tuberculosis subsp*.*tuberculosis,* slow growing non-pathogenic *Mycobacterium tuberculosis*; Strain H37Ra and fast growing non-pathogenic *Mycobacterium smegmatis*. The results of the minimum inhibitory concentration (MIC) values of the crude extracts are shown in Table 1 below. In the present study, the leaves from *S. torvum* displayed the best *in vitro* activity against all the mycobacterial strains tested (Table 1).

The cytotoxic potential of the crude plant extracts were also evaluated against MRC-5 human fetal lung fibroblast cell line. The median cytotoxic concentration (CC_50_ in μg/mL) determinations and selectivity indices (SI) are shown in Table 2 below. *S. torvum* leaves had the highest CC_50_ value and the best SI index of 31.25 (Table 2).

The present study was further extended to assess the best predictor of activity against pathogenic *M. tuberculosis* amongst the non-pathogenic mycobacterial strains used in the current study. The correlation coefficients (r) values obtained are presented in Table 3. *M. tuberculosis*; strain H37Ra was the best predictor of activity against the pathogenic *M. tuberculosis* (correlation coefficient=0.7723) (Table 3).

Phytochemicals are usually classified as antimicrobials on the basis of susceptibility tests that produce minimum inhibitory concentrations (MICs) in the range of 100 to 1000 µg/mL (Kuete, 2010). Activity of crude extracts is considered to be significant if MIC values are below 100 µg/mL, moderate when 100<MIC<625 µg/mL or low when MIC>625 µg/mL (Kuete, 2010). Therefore, the activity recorded from the leaves of *S. torvum* of 156.3 µg/mL against *M. tuberculosis; strain* H37Ra can be considered to be moderate. Alternative criteria has been described by Fabry et al. (1998), which consider extracts having MIC values below 8000 µg/mL to have noteworthy antimicrobial activity. Under these less stringent criteria, and considering the fact that the medicinal plants tested in the current study are used traditionally to treat upper and lower respiratory tract related conditions, the activity recorded from the tested crude extracts could be considered important.

The leaves of *Solanum torvum* are used in Ghanaian ethnomedicine to treat coughs and tuberculosis (Nguta et al., 2015a). The current study has observed moderate *in vitro* antimycobacterial activity from the leaves of *S. torvum* against the slow growing nonpathogenic mycobacterial strain, *M. tuberculosis; strain* H37Ra. Interestingly, the leaves also inhibited the growth of the pathogenic *M. tuberculosis* strain at 1250 µg/mL. These findings validate in part the ethnoparmacological use of the leaves by local communities in the eastern and greater regions of Accra to treat disease conditions whose symptoms closely resemble tuberculosis, and hence the local communities can be trusted with the information they shared with us in the course of the study. The current data provides evidence that the screened leaves are a potential source of molecules against tuberculosis. Moderate antimycobacterial activity against the pathogenic laboratory reference strain H37Rv by hydromethanolic fruit extracts from *S. torvum* has been reported in Malaysia (Mohamad et al., 2011), further supporting current findings, while adding more weight to the ethnopharmacological utilization of the leaves in traditional medicinal systems. It is worth noting that, the extracts in the current study were hydroethanolic in nature, suggesting that the phytoconstituents responsible for the antimycobacterial activity from both the leaves and the fruits of *S. torvum* are possibly in the hydroethanolic fraction. These observations from different geographical zones render more support to the ethnobotanical use of different parts of *S. torvum* in management of tuberculosis and call for intensified studies focusing on isolation and elucidation of anti-tb bioactive constituents from the medicinal plant. Antimicrobial activities of the leaf and fruit of this plant have been reported (Ajaiyeoba, 1999, Wiart et al., 2004), supporting observations from the current study. Methy caffeate, isolated from the unripe fruits of *S. torvum* inhibited the growth of *M. tuberculosis* (H37Rv) at a dose of 8 μg/mL (Chandrasekhar et al., 2012), making the compound a potential target for drug development against *M. tuberculosis*. This report further supports the antimycobacterial potential of *S. torvum*, throwing more weight to the current findings and calling for more studies aimed at developing a new class of drugs against tuberculosis from different parts of *S. torvum*. To the best of our knowledge, the antimycobacterial activity from the leaves of *S. torvum* is reported for the first time by the current study. The hydroethanolic extract from the leaves of *S. torvum* was found to be safe in MRC-5 fetal fibroblast lung cells, with a wide margin of safety as indicated by the observed selectivity index (SI) (Table 2), thus supporting the traditional use of the leaves for the management of lower respiratory tract conditions. The observed wide therapeutic window further supports the urgent need for more studies aimed at identifying a safe and efficacious novel class of compounds with activity against the pathogenic *M. tuberculosis*. The cytotoxic effects of two compounds (Solanolactosdie A, Solanolactoside B) isolated from the aerial parts of *S. torvum* were not significant (Zubaida et al., 2013), supporting the safety findings from the leaves of *S. torvum* observed in the current study. Further bioassay guided studies are ongoing in our laboratories, with the aim of isolating and elucidating the structure of bioactive molecules from the leaves of *S. torvum.*

The rhizomes of *Z. officinale* are traditionally used to treat coughs and related lower respiratory tract conditions by the local communities of eastern and greater regions of Ghana (Nguta et al., 2015a). The observed inhibitory effects, is a validation to the medicinal value of the rhizomes from *Z. officinale.* Ginger (*Z. officinale*) extract (10 mg/kg) administered intraperitoneally has been shown to possess a dose-dependent antimicrobial activity against *Pseudomonas aeruginosa*, *Salmonella typhimurium*, *E. coli* and *Candida albicans*. In addition, out of 29 plant extracts screened, ginger extract was found to have the broadest range of anti-fungal activity measured either by the fungi inhibited or as the average diameter of the zones of inhibition and was the only crude extract that was active against Rhizopus sp., an organism that was not inhibited by any of the other plant extracts tested or by the anti-fungal agent ketoconazole or berberine (Ali et al., 2008). This observations are in agreement with the current findings since the crude extract was able to inhibit the growth of the non-pathogenic mycobacterial strain, *M. tuberculosis* strain H37Ra at a dose of 2500 µg/mL. It is plausible to hypothesize that the low activity recorded in the present study could be due to the presence of the bioactive antimycobacterial compounds in low concentrations from the investigated rhizomes. Furthermore, the rhizomes are used as decoctions or concoctions in traditional medicinal systems. The observed toxicity and low selectivity index is a pointer that *Z. officinale* may not be a safe medicinal plant for use against tuberculosis. The hydroethanolic leaf extract from *A. vera* inhibited the growth of *Mycobacterium tuberculosis subsp*.*tuberculosis, M. tuberculosis;* strain H37Ra and *M. smegmatis* at MIC values of 10,000, 2500 and 5000 µg/mL respectively. The antimycobacterial potential of the leaves of *A. vera* is supported by earlier observations of antimycobacterial activity (Gupta et al., 2010) against multi-drug resistant (MDR) strains and laboratory reference strain H37Rv while screening aqueous extracts of *Aloe vera* using Lowenstein–Jensen medium and Middlebroook 7H9 broth. However, the high cytotoxicity against MRC-5 fibroblast cell line and low selectivity index is a signal to the fact that the investigated crude extract may not be safe for use as a remedy against tuberculosis. The leaves of *D. rotundifolia* are traditionally used by different Ghanaian communities to treat coughs and lower respiratory tract conditions. The observed antimycobacterial activity against pathogenic *Mycobacterium tuberculosis subsp*.*tuberculosis* and non-pathogenic *M. smegmatis* at MIC values of 10,000 and 5000 µg/mL respectively validates the ethnopharmacological use of the plant by the Ghanaian communities. However, the leaves may not make safe preparations against tuberculosis since they have a narrow therapeutic window and are highly toxic to the MRC-5 cell line, calling for further studies to evaluate their *in vivo* toxicity. To the best of our knowledge, the present study reports for the first time the *in vitro* antimycobacterial activity and cytotoxicity of *D. rotundifolia. Chenopodium ambrosoides* leaves are commonly used against chronic coughs and related low respiratory tract conditions in Ghanaian ethnomedicine (Nguta et al., 2015a), hence the current observations validates the ethnopharmacological use of the said plant. In the current study, hydroethanolic extracts from the leaves inhibited *Mycobacterium tuberculosis subsp*.*tuberculosis and M. tuberculosis;* strain H37Ra at MIC values of 10,000 and 5000 µg/mL respectively. The current findings are in agreement with earlier observations of *in vitro* antimycobacterial activity of acetonic leaf extracts against drug sensitive and resistant strains of *M. tuberculosis* (Lall and Meyer, 1999). The cytotoxic activity exhibited against the MRC-5 cell line calls for further *in vivo* evaluation of the leaves for safety. Majority of natural product research scientists with interests in anti-tb drug discovery have no access to a BSL-3 laboratory (Nguta et al., 2015b), hence the search for a non-pathogenic mycobacterial strain with a similar drug susceptibility profile similar to that of the pathogenic *M. tuberculosis* is a priority. Such information will enable scientists in laboratories without a BSL-3 facility to equally contribute to drug discovery efforts against tuberculosis. The current study observed *M. tuberculosis;* strain H37Ra to be the best predictor of activity of the evaluated crude extracts against the pathogenic *Mycobacterium tuberculosis subsp*.*tuberculosis,* hence could be used in a BSL-2 laboratory to investigate natural products for activity against the pathogenic *M. tuberculosis*.

## 4 Conclusion

The results from the present study are quite exciting, taking into consideration the medical importance of the studied mycobacterial strains. The observed results justify the screening of medicinal plants traditionally used against respiratory tract conditions to find a cure against tuberculosis. These data provides evidence that the hydroethanolic (80% ethanol) leaf extract from the leaves of *Solanum torvum* have moderate activity against the tested mycobacterial strains and a potential cure for tuberculosis. Hence further bioassay guided studies are ongoing in our laboratories with an aim of isolating an efficacious and a safe antimycobacterial compound.

## Figures and Tables

**Table 1:** Minimum inhibitory concentration (MIC in µg/mL) values of hydro-ethanolic extracts from selected medicinal plants.

Plant species/family/voucher specimen number	Part used	*M. smegmatis*	*M. tb*; strain H37*Ra*	*M. tb* H37Rv
*Solanum torvum* SW./ Solanaceae/JM01	Leaves	2500	156.3	1250
*Zingiber officinale* roscoe*/Zingiberaceae/*JM02	Rhizomes	10,000	2500	10,000
*Aloe vera* var. *barbadensis/*Xanthorrhoeaceae/JM03	Leaves	10,000	2500	5000
*Dissotis rotundifolia* (Sm.) Triana/Melastomataceae/JM04	Leaves	10,000	>10,000	5000
*Chenopodium ambrosioides* L./Amaranthaceae/JM05	Leaves	10,000	5000	>10,000
Isoniazid (INH)	–	2	0.08	0.03
Rifampicin (RIF)	–	4	0.08	0.08
Ethambutol (EMB)	–	0.06	0.50	2

Experiments were carried out in triplicate and results are expressed as means of three replicate experiments. All the crude extracts were dissolved in distilled water. Essential anti-tb drugs were prepared according to manufacturer’s instructions. Tested concentration range for crude extracts: 19.5–10,000 µg/mL; tested concentration range for positive control drugs: INH: 0.025–1.6 µg/mL; RIF: 0.25–16 µg/mL; EMB: 0.25–16 µg/mL. (-): pure compounds tested.

**Table 2:** Cytotoxicity (CC_50_ in µg/mL) of crude extracts in MRC-5 lung fibroblast cells and selectivity index (SI) values in the three tested Mycobacterial strains.

Plant species/family	CC_50_	Selectivity index (SI) values		
		*M. smegmatis*	*M. tb*; strain H37*Ra*	*M. tb* H37Rv

*Solanum torvum* SW./Solanaceae	31,250	12.5	199.94	25
*Zingiber officinale* roscoe/Zingiberaceae	3500	0.35	1.4	0.35
*Aloe vera* var. *barbadensis/*Xanthorrhoeaceae	3200	0.32	1.28	0.64
*Dissotis rotundifolia* (Sm.) Triana/Melastomataceae	600	0.06	nd(MIC>10)	0.12
*Chenopodium ambrosioides* L./Amaranthaceae	4800	0.48	0.96	nd (MIC>10)
Isoniazid	12.5	6.25	156.25	416.7

Experiments were carried out in triplicate and results are expressed as means of three replicate experiments. All the crude extracts were dissolved in distilled water. nd: not determined; MIC>10,000 µg/mL: crude extracts were not active at up to 10,000 µg/mL. Voucher specimen numbers are indicated in Table 1.

**Table 3:** Pearson’s correlation coefficients (r) between MIC values of the three *mycobacterium* strains used.

	*Ms*	*M. tb* H37*Ra*	*M. tb*H37Rv
*Ms*	1		
*M. tb* H37*Ra*	0.9759	1	
*M. tb*H37Rv	**0.7698**	**0.7723**	**1**

*Ms*: *Mycobacterium smegmatis; M. tb* H37Ra: *Mycobacterium tuberculosis*; Strain H37Ra; *M. tb*H37Rv: *Mycobacterium tuberculosis subsp*.*tuberculosis* Values in bold indicate the correlation between the non-pathogenic Mycobacterial strains and the pathogenic *Mycobacterium tuberculosis subsp*.*tuberculosis.*
